# Ethics in Trichology

**DOI:** 10.4103/0974-7753.66912

**Published:** 2010

**Authors:** Parvathi Padmanabhan

**Affiliations:** Dermatologists, Chennai, India

**Keywords:** Ethics, trichology, dermatology

## Abstract

Today, trichology as a science is offered by reputed institutes worldwide with a planned curriculum of theory and clinical exposure. Non-medical trichologists, with rare exceptions, continue to lure the public with unscientific methods. A qualified dermatologist equipped with knowledge of hair biology is undoubtedly the most competent to deal with hair problems. We are all indisputably governed by a basic code of medical ethics as we are doctors first and last. Hence, a dermatologist/trichologist cannot have another set of ethics. Having said that, we cannot discount the fact that there are special clinical situations where guidelines already exist or need to be established with compliance to the base code of ethics.

## INTRODUCTION

Ethics can be defined as “a set of principles concerning proper conduct.”[1] Normally, we live our lives and operate our business, our practice or research based on our own moral code without consciously realizing that we have a moral code that we live by. Our moral code is essentially our sense of what is right and what is wrong or what is acceptable and what is not acceptable. This code cannot be the same for everyone because we all think differently and we all have different upbringings, backgrounds and experiences. Thus, our own definition of ethics may be different from that of others.

However, certain ground rules remain non-negotiable. As a touchstone to ethical action, we ask ourselves, are we conducting ourselves in a manner that we can be proud of and not want to hide or be ashamed of our actions?

Medical ethics is an extension of general ethics, applied to doctors and special situations, duly codified. This is well elucidated by the “four principles plus scope” approach, which provides a basic prima facie moral commitment — *respect for autonomy, beneficence, non-maleficence and justice* — plus concern for their scope of application.[2] It offers a common, basic moral analytical framework and a common, basic moral language and can help doctors make decisions when reflecting on moral issues that arise at work.

Humans are blessed with a free will to make choices. The choices that we make, both in our personal and professional lives, determine our code of ethics — the choice of our goals and the methods we use to get there.

Alice came to a fork in the road. “Which road do I take?” she asked.

“Where do you want to go?” responded the Cheshire cat.

“I don’t know,” Alice answered.

“Then,” said the cat, “it doesn’t matter.”

Alice in wonderland — Lewis Caroll

It does matter to us where we need to go. All of us confront situations from time to time in our professional lives, when we have to choose our path of conduct. True, no one may be looking over our shoulder, no one to truly police, enforce or penalize us when we jump a red light. *To take the ethical path, which is often not very easy, requires courage of conviction and a clear vision of our goals.*

Every once in a while we need to pause, introspect and course correct. In this internal process, a learning curve of ethics follows. However, the same can be learnt from the outside — wherein the question arises — can ethics be learnt by being taught or is it best imbibed by being inspired by others who can be our role model.

Socrates, the philosopher, thought ethics can be taught, almost 2,500 years ago.[3] He believed that no one errs intentionally. This means that whenever we do something wrong, including something morally wrong, it is out of ignorance rather than evil. Socrates’ position was clear: *Ethics consists of knowing what we ought to do, and such knowledge can be taught.*

We have seen the recent financial meltdown in the United States as an example and consequence of human greed and unethical practices. To mitigate this, business schools have reinforced Business Ethics as an integral part of their curriculum today.

It is true that we learn best from an example set by others. A person’s ethics can change for the better, but only if the person is motivated to change them. The best source of ethics is from a mentor, a role model who inspires you to follow. There is a Buddhist saying, “If the student is ready, the teacher will appear,” and if you get an inspirational teacher, you are indeed blessed!

Clearly, knowledge of ethics is useful only when it produces a transformation within us and reflects in our actions. A strong conviction helps overcome the obstacles of greed, desire for fame and power, which deter the progress in the knowledge-action path.

## ON THE MEDICAL FRONT…

Ethical depravity amongst doctors exists in many forms. One form of such is a mental disorder, Profit-Driven Medical Disorder (PDMD[4] Doctors who are unethical may be actually suffering from PDMD, which affects thousands of doctors in all specialties and dermatology, with its income-dense super specialties of cosmetology, trichology, etc., is no exception!

The Coalition to Raise Awareness of PDMD is committed to helping these doctors.

Although all of us deserve to be well-compensated for our skills and dedication, victims of PDMD feel an overwhelming urge for profit and prestige, which improperly influences their research and patient care. Doctors with PDMD have no control over their behaviors and most suffer in silence and secrecy.

A PDMD victim regularly accepts money or expensive gifts from pharmaceutical companies, serves as a paid speaker for specific drugs and treatments, conducts big pharma-funded clinical research with intent to manipulate, suppresses or fabricate clinical trial data to help companies sell more drugs and puts his/her name on ghostwritten papers or sell his/her raw data to commercial organizations.

The probable causes of PDMD are:

1. Like other mental illnesses, it reflects biological dysfunctions in the brain.
2. Family background and life experiences may be involved.
3. Certain genes predispose to PDMD.
4. When doctors feel that they are not receiving the level of respect or compensation owed to them, they become stressed, which may result in PDMD.

Life events such as purchasing a home or automobile or getting married and having children can also trigger the symptoms.

## OUR RESPONSIBILITIES

As ethical dermatologists/trichologists, we should work proactively at eliminating quackery in trichology by ensuring the following:

1. We must equip ourselves with the necessary knowledge and skill sets by working in tandem with hair biologists to address all hair problems and update continually.
2. We ought to spend time with the patient, explaining the etiology, pathogenesis, treatment options and expected outcome of treatments and use patient education pamphlets.
3. We should raise public awareness about charlatans.
4. In today’s world, there is a distinct need to instill dermatology graduates with a clear code of ethics at the post-graduate curriculum level and reinforce the same in practice through exposure to senior consultants who will remain as role models for the younger generation.

Of all the rules that govern our conduct, none “completes” the spirit of the process as comprehensively as the “Golden Rule of Ethics of Reciprocity.”

“Do onto others as you would wish them do onto you.”
